# Suppressor T cells in BCG-treated mice interfere with an in vivo specific antitumoral immune response.

**DOI:** 10.1038/bjc.1984.119

**Published:** 1984-06

**Authors:** B. Payelle, M. Brulay-Rosset, M. F. Poupon, G. Lespinats

## Abstract

The interference by BCG in the induction and expression of a specific antitumoral immune reaction was studied in B6 mice, using the in vivo Winn assay and also active immunization. T cells immunized against MCA-induced fibrosarcoma (MC B6-1) transferred together with the tumour cells protected the syngeneic host against tumour take. Pretreatment of normal B6 mice with moderate or high doses of BCG prevented the development of a protective immune response after immunization. Moreover, a single dose of 1 mg, or 2 doses of 0.01 mg BCG, completely eliminated an established antitumour immunity. Suppressor cells are involved in the BCG-induced inhibitory effect; they interfered (1) with the expression of the antitumour response, since their addition to immune T cells in the Winn test resulted in decreased protection and (2) with the induction of the antitumour response, since injection of spleen cells from BCG-treated mice (BCG SpC) into normal mice before immunization inhibited the development of immunity. Treatment of BCG SpC with anti Thy 1.2 and anti Lyt 1.2 antibodies plus complement before injection into normal mice significantly decreased the suppressive activity, showing that the suppressor cells induced by BCG are T cells expressing the Lyt 1+ phenotype. The partial increase in protection obtained after IL-2 administration to BCG-treated mice suggests that the suppressive action of BCG SpC on the IL-2 producing capacity of helper T cells is only one of a number of possible mechanisms of T-cell-mediated suppression.


Br. J. Cancer (1984), 49, 759–768

### Suppressor T cells in BCG-treated mice interfere with an *in vivo* specific antitumoral immune response

B. Payelle[1], M. Brulay-Rosset[2], M.F. Poupon[1] & G. Lespinats[1]

[1]*Laboratoire d'Immunobiologie – Institut de Recherches Scientifiques sur le Cancer, BP 8, 94800 Villejuif,* [2]*Institut de Cancérologie et d'Immunogénétique, Hôpital Paul-Brousse, 14 avenue Paul-Vaillant-Couturier, 94804 Villejuif Cedex, France.*

**Summary** The interference by BCG in the induction and expression of a specific antitumoral immune reaction was studied in B6 mice, using the *in vivo* Winn assay and also active immunization. T cells immunized against MCA-induced fibrosarcoma (MC B6-1) transferred together with the tumour cells protected the syngeneic host against tumour take. Pretreatment of normal B6 mice with moderate or high doses of BCG prevented the development of a protective immune response after immunization. Moreover, a single dose of 1 mg, or 2 doses of 0.01 mg BCG, completely eliminated an established antitumour immunity. Suppressor cells are involved in the BCG-induced inhibitory effect; they interfered (1) with the expression of the antitumour response, since their addition to immune T cells in the Winn test resulted in decreased protection and (2) with the induction of the antitumour response, since injection of spleen cells from BCG-treated mice (BCG SpC) into normal mice before immunization inhibited the development of immunity. Treatment of BCG SpC with anti Thy 1.2 and anti Lyt 1.2 antibodies plus complement before injection into normal mice significantly decreased the suppressive activity, showing that the suppressor cells induced by BCG are T cells expressing the Lyt $1^+$ phenotype. The partial increase in protection obtained after IL-2 administration to BCG-treated mice suggests that the suppressive action of BCG SpC on the IL-2 producing capacity of helper T cells is only one of a number of possible mechanisms of T-cell-mediated suppression.

BCG treatment has been shown to induce tumour regression by stimulating cell-mediated immunity (Old *et al.*, 1959; Zbar *et al.*, 1970; Baldwin *et al.*, 1976). However, numerous studies have established that treatment with high doses of BCG provoked suppressive effects, revealed by inhibition of reactions such as lymphocyte proliferation (Turcotte *et al.*, 1978), delayed-type hypersensitivity (Watson & Collins, 1980; Florentin *et al.*, 1976), T cell cytotoxicity developed by mixed lymphocyte culture (Klimpel & Henney, 1978; Davies & Sabbadini, 1977), or antibody production (Schrier *et al.*, 1980; Kitamura *et al.*, 1976). Recently, Kendall & Sabbadini (1981) demonstrated that BCG treatment could reduce the production of helper factors (IL-2) capable of stimulating T cell growth, and Klimpel *et al.* (1979) found that this deficit in IL-2 synthesis by helper T cells could be overcome by the *in vitro* addition of exogenous IL-2.

We have used the test of adoptive transfer of immunity (Winn, 1961) to study the effects of BCG treatment on the induction of an *in vivo* T cell-mediated specific immune response to tumour antigen. This model has allowed us to study the interactions of BCG at different phases (induction and expression) of the T cell-mediated protective mechanism against a chemically-induced tumour. We have previously demonstrated that B6 mice can be immunized against MC B6-1 tumour cells, a MCA-induced fibrosarcoma, and that immune T cells transferred together with MC B6-1 tumour cells protected the syngeneic host against tumour take. This control of tumour growth *in vivo* was the result of a complex immunologic mechanism involving cooperation between immunized T lymphocytes and a radio-resistant non T cell of host origin (Poupon *et al.*, 1981).

In the present study we report that BCG at high doses, injected before immunization, can completely prevent the generation of specific protective T cells, or even abrogate an already established antitumour immunity. We demonstrate the presence of suppressor cells in the BCG-treated animals, since transfer of BCG spleen cells to normal mice inhibited the development of active immunity. Also, the addition of lymphoid cells from BCG-treated mice to immune T cells before transfer to recipient mice abrogated passive protection against tumour growth. These suppressor cells belong to the T cell subpopulation expressing the Lyt 1 phenotype. An attempt to eliminate the suppressive effect of BCG treatment by administration of a lymphocyte helper factor (IL-2) had only a limited success.

Correspondence: M. Brulay-Rosset
Received 19 October 1983; accepted 1 March 1984.

© The Macmillan Press Ltd., 1984

### Materials and methods

### *Mice*

Female 8- to 12-week-old C57BL/6 (B6) H-$2^b$ haplotype mice were obtained from Bom Holtgaard Laboratories (Denmark).

### *BCG*

From Pasteur Institute, containing $7 \times 10^6$ viable units $mg^{-1}$, was administered i.v. to B6 mice at different doses.

### *Tumour*

The MC B6-1 fibrosarcoma was originally induced by the s.c. injection of 2 mg of methylcholanthrene (MCA) into female B6 mice, and was then serially transplanted in syngeneic females. MC B6-1 was used between the 19th and 24th passages, at a routine dose of $10^4$ cells, which produced a palpable tumour within 7–14 days in 96% of mice (calculated on >200 mice). Tumour cells were dissociated from the solid tumour by trypsinization at 37°C for 90 min. After washing, the number of viable cells, determined by trypan blue exclusion, always exceeded 90%.

### *Immunizations*

Two protocols of tumour immunization were used, one with living MC B6-1 tumour cells, the other with AB2 hybrid tumour cells, which are semi-allogeneic somatic hybrid cells derived from the fusion of a MC B6-1 tumour cell and a A9 cell (a fibroblastic cell of C3H origin).

(i) *Immunization with MC B6-1 tumour cells* The immunization protocol has been described previously (Poupon *et al.*, 1979). B6 mice received an initial s.c. injection of $5 \times 10^5$ cells obtained from a solid tumour. After 10 days, the resulting tumour was surgically removed. The mice then received challenges of tumour cells at 21-day intervals, and at progressively increasing doses; any tumour take was followed by surgical removal. After the 5th challenge, mice were able to reject a dose of $10^5$ tumour cells and were thus considered immune.

(ii) *Immunization with AB2 hybrid tumour cells* This immunization protocol, described previously (Payelle *et al.*, 1981), used a single hybrid cell inoculation. These AB2 hybrid cells did not produce tumours when injected into B6 mice because of the presence of the H-$2^k$ histocompatibility antigens of the allogeneic A9 parental cell. They did, however, induce antitumour immunity against the syngeneic MC B6-1 parental tumour cell. AB2 hybrid cells were maintained in culture and injected as a single dose of $5 \times 10^6$ i.p. Eight days after AB2 inoculation, mice were able to reject a graft of $10^4$ MC B6-1 tumour cells and were, therefore, also considered immune.

### *Preparation of T cell suspensions*

As previously described (Poupon *et al.*, 1979), spleens were removed from mice, dissociated, filtered through a stainless steel sieve and washed in MEM. To separate the T cell-enriched population, $10^8$ total spleen cells in 2 ml MEM plus 10% FCS were placed on a column of 0.2 g nylon wool (LP-1 leucopak leukocyte filter; Fenwall Laboratories, Morton Grove, IL), incubated for 60 min at 37°C, and rinsed with 15 ml MEM 10% FCS at 37°C (Julius *et al.*, 1973). The percentage of harvested cells varied between 20 and 30%.

### *Procedure for Winn assay*

MC B6-1 tumour cells ($10^4$) were mixed with immunized T cells in a volume of 0.2 ml of MEM at various T cell-tumour ratios as indicated in the text. Each group consisted of 8 or 10 mice which were checked every 2 or 3 days. The number of tumour-bearing animals was recorded and the growth rate was followed for 30 days.

### *Test of resistance*

AB2 hybrid tumour cell immunized mice, previously treated by BCG or non-treated (controls) received an injection of $10^4$ MC B6-1 tumour cells s.c. in the flank. Groups of 10 mice were identically treated. After one week, the mice were checked every 2 to 3 days. The number of tumour-bearing animals was recorded.

### *Transfer system*

Spleen cells from normal (N-SpC) or BCG-treated (BCG SpC) mice were injected i.v. into syngeneic hosts as populations that were either unfractionated ($5 \times 10^7$ cells/mouse), anti Thy 1.2 treated ($5 \times 10^7$ treated cells/mouse), or separated by passage through a nylon-wool column ($2 \times 10^7$ cells/mouse). In some experiments the T cell enriched population ($2 \times 10^7$ nylon non-adherent cells/mouse) were treated with Lyt 1.2 or Lyt 2.2 antisera + C before administration. The mice receiving the various populations of BCG SpC received $5 \times 10^6$ AB2 hybrid cells 2 h later.

### *Treatment of spleen cells with monoclonal anti Thy 1.2 antibody and complement*

Spleen cells ($30 \times 10^6$) were placed for 1 h in an ice bath with 0.5 ml of a $10^{-3}$ dilution of monoclonal anti Thy 1.2 antibody (New England Nuclear). The cells were then washed and resuspended in 1.5 ml of a 1:25 dilution of rabbit complement (Cedarlane Laboratories Ltd, Canada) for 45 min at 37°C in a water bath with agitation.

*Treatment of splenic T cells with monoclonal anti Lyt 1.2 or anti Lyt 2.2 antibody and complement*

Splenic T cells obtained after passage through a nylon wool column ($3 \times 10^7$) were placed for 1 h in 0.5 ml monoclonal anti Lyt 1.2 or anti Lyt 2.2 antibody (New England Nuclear), at a 1:100 and 1:50 dilution respectively. The cells were then washed and resuspended in 1.5 ml of a 1:25 dilution of rabbit complement (Cedarlane Laboratories) for 45 min at 37°C in a water bath with agitation.

*IL-2*

IL-2 was a generous gift of Dr Chaouat, IRSC, Villejuif, France. It was produced by stimulating the EL4 C-16 cell line for 24 h with phorbol-12-myristate acetate. This preparation contained 400 u ml$^{-1}$. IL-2 was adninistered i.p. daily to B6 mice at a dose of 20 u four days before, and 4 days after, AB2 hybrid tumour cell immunization on Day 0. In this experiment, mice received BCG i.v. on Day 14 at a dose of 1 mg.

*Enumeration of BCG spleen colonies*

Spleens were prepared in sterile distilled water and serial dilutions were made from 1/50 to 1/40000. One hundred µl of every suspension was inoculated into tubes containing Jensen medium. The number of colonies was enumerated 3 weeks later for each dilution and the total number of colonies per spleen was calculated.

*Statistical evaluation*

Statistical significance of differences between groups was calculated using Fisher's exact test.

### Results

*Effect of BCG treatment on immunization against tumour cells*

*Active immunization* B6 mice received i.p. $2 \times 10^6$ AB2 hybrid tumour cells 14 days after BCG administration at different doses. Eight days later, a s.c. challenge of $10^4$ MC B6-1 cells was given to these mice. The effect of BCG on the level of immunity produced by AB2 hybrid tumour cells was evaluated by the number of mice developing tumours in each group. Treatment with 1 mg BCG led to a significantly decreased ($P<0.01$) efficiency of the immunization, resulting in a loss of resistance, even at the lowest doses ($P=0.03$) (Table I).

**Table I** Effect of BCG pretreatment of immunized mice on their resistance to tumour challenge

| *Dose of BCG*[a] (*mg*) | *No. of mice with tumour*[b] / *Total no. of mice* | *P values*[c] |
|---|---|---|
| 0 | 6/16 | — |
| 0.05 | 12/15 | $P=0.03$ |
| 1 | 16/16 | $P<0.001$ |

[a]BCG was injected i.v. on day −14, AB2 cells i.p. on Day 0, and MC B6-1 tumour s.c. on Day 7.
[b]Recorded on Day 30.
[c]Compared with control group receiving no BCG treatment.

*Adoptive immunization* B6 mice received $5 \times 10^6$ AB2 hybrid tumour cells i.p. 14 days after administration of 0.05 mg, 1 mg, or 5 mg BCG per mouse. Spleens were excised 7 days later and T cells were enriched by passage through a nylon wool column. Different numbers of T lymphocytes from N-SpC or BCG-SpC were mixed with $10^4$ tumour cells (MC B6-1 fibrosarcoma) and injected s.c. into syngeneic hosts. Table II shows that protection against tumour take was obtained by transfer of T lymphocytes originating from non BCG-treated immunized mice, at both splenic T cell-to-tumour cell ratios (300:1 and 150:1). When BCG was administered before immunization, the number of animals with tumours increased as the dose of BCG increased. For the dose of 5 mg, no protection at all was observed ($P<0.01$). At the 300:1 and 150:1

**Table II** Effect of BCG pretreatment of immunized mice on the protection of adoptively transferred mice

| *Dose of BCG*[a] (*mg*) | *No. of mice with tumour*[b] / *Total no. of mice* | |
|---|---|---|
| | *300:1*[c] | *150:1* |
| 0 | 8/18 | 8/20 |
| 0.05 | 4/10 | 4/10 |
| 1 | 12/18 | 18/20[d] |
| 5 | 18/20[d] | 18/20[d] |

[a]Immunization i.p. with AB2 cells was performed 14 days after i.v. BCG or control injection. Seven days later immune T spleen cells were mixed with tumour cells and transferred s.c. to normal recipient mice.
[b]Recorded on Day 30.
[c]Immune T spleen cell:tumour cell ratio.
[d]Significantly different ($P<0.01$) from control group receiving no BCG treatment.

lymphocyte:tumour cell ratios the lowest dose (0.05 mg BCG) allowed a good protection.

*Influence of BCG on an established antitumour immunity*

In this experiment B6 mice were immunized by progressively increasing the challenge dose of live MC B6-1 cells and excising any subsequent tumours. After 5 tumour cell challenges (protocol under Table III), all mice were able to reject a tumour graft of $1 \times 10^5$ cells. At that time, immunized mice received 0.01 mg or 1 mg BCG i.v. and a $10^5$ MC B6-1 tumour cell challenge was performed 12 days later. After an additional 10 days, immune splenic T cells from the different groups of animals were transferred to normal recipient mice together with MC B6-1 cells in a Winn assay. Results giving the percentage of tumour-bearing recipient mice are presented in Table III. Passive protection against tumour growth was improved by 0.01 mg BCG administration (78% *versus* 50% in control mice at 600:1 lymphocyte to tumour cell ratio) given before the last tumour cell challenge. In contrast, an injection of 1 mg BCG abrogated the passive transfer of immunity (100% tumour take) ($P=0.05$).

**Table III** Effect of BCG on pre-existing antitumour immunity

| *Dose of BCG*[a] *(mg)* | *No. of mice with tumour*[b] / *Total no. of mice* 600:1[c] | *300:1* |
|---|---|---|
| 0 | 5/10 | 8/8 |
| 0.01 | 2/9 | 5/8 |
| 1 | 8/8[d] | 7/8 |

[a]Immune mice received 10 days after the 5th challenge of $10^5$ live tumour cells s.c., either 0.1 ml PBS i.v., or 0.01 mg BCG i.v., or 1 mg BCG i.v. Twelve days later all the mice received a 6th challenge of $10^5$ live MC B6-1 tumour cells s.c. Winn test was performed 10 days later.
[b]Recorded Day 30.
[c]Immune T spleen cell:tumour cell ratio.
[d]$P=0.05$ compared with control group receiving no BCG treatment.

In a second experiment, groups of mice were prepared as described in the protocol under Table IV. All immunized mice having received a second injection of 1 mg BCG developed tumours, therefore were not rechallenged with MC B6-1 tumour cells, and died rapidly. No tumours were observed either in the control immune group receiving PBS and MC B6-1 cells, or in the group receiving 0.01 mg BCG+MC B6-1 cells. In order to determine whether the efficiency of immunization mediated by T cells could be further increased, a second BCG injection of 0.01 mg was given to these mice followed by MC B6-1 inoculation 12 days later (protocol under Table IV). Splenic T cells were then transferred to syngeneic hosts mixed with tumour cells. We observed that 87% of the mice were still protected against tumour growth in the control group, but that the second injection of 0.01 mg BCG significantly ($P<0.05$) abrogated the protective effect of previous immunization (Table IV). From both of these results, it may be concluded that high doses of BCG not only reduced the efficiency of immunization, but also abrogated an established immunity, Moreover, while a small dose was able to increase the efficiency of immune T cells, its repetition led to the same effect as one high dose.

**Table IV** Effect of repetition of BCG on a pre-existing immunity

| *Dose of BCG*[a] *(mg)* | *No. of mice with tumour*[b] / *Total no. of mice* 600:1[c] | *300:1* |
|---|---|---|
| 0 | 1/8 | 1/8 |
| 0.01 | 6/8[e] | 8/8[e] |
| 1[d] | — | — |

[a]Immune mice received, 10 days after a 5th challenge of $10^5$ live tumour cells s.c., either 0.1 ml PBS i.v., 0.01 mg BCG i.v., or 1 mg BCG i.v. Twelve days later all the mice received a 6th challenge of $10^5$ live tumour cells s.c. Each group then received respectively 0.01 ml PBS, 0.01 mg BCG, or 1 mg BCG 10 days after the 6th tumour cell challenge. A 7th tumour cell challenge was performed 12 days later followed by Winn test 10 days later.
[b]Recorded on Day 30.
[c]Immune T spleen cell:tumour cell ratio.
[d]These mice were not tested since they had growing tumours and died rapidly after the second injection of BCG.
[e]Significantly different ($P<0.05$) from control group receiving no BCG treatment.

Since the BCG used is a live vaccine, the number of colonies in the spleen of mice injected with 0.05 mg became similar to that observed with 1 mg at Day 14, although with a delay of 21–28 days (Figure 1). Therefore, the application of a small dose such as 0.01 mg BCG twice at a 3 week interval may give rise to a similar bacterial load to that obtained with an inoculum of 1 mg BCG 14 days before. This may lead to the deterioration of the protective effect of cytotoxic T cells against tumour growth.

**Figure 1** Number of BCG colonies in the spleen of mice treated i.v. with 0.05 mg (——), 0.2 mg (—-—), or 1 mg (----) BCG as a function of time after i.v. administration.

*Evidence for suppressor cells in BCG-treated mice*

In order to demonstrate a suppressive activity mediated by cells present among BCG-SpC, a cell mixture experiment was performed as follows: $10^7$ spleen cells from 0.05 mg or 1 mg BCG-treated mice were added to $4 \times 10^6$ immune T cells and $10^4$ tumour cells. This mixture was transferred to syngeneic hosts. The control group consisted of mice receiving N-SpC with immune T cells and tumour cells (Table V). We observed some degree of protection in the control group (6/18 mice developed tumours). The addition of $10^7$ 0.05 mg BCG SpC did not modify the antitumour protection (Table V). In contrast, the addition of $10^7$ 1 mg BCG-SpC resulted in a significant ($P<0.02$) decreased protection (14/18 mice with tumours). These observations demonstrated that high dose BCG induced suppressor cells that act during the effector stage of the antitumour immune reaction.

A second approach was to determine the effect of these cells on the development of antitumour immunity. We injected i.v. N-SpC or 1 mg BCG-SpC into syngeneic B6 hosts which received AB2 hybrid cells 2 h later. Eight days later, splenic T cells from these i.v. injected immunized mice were tested for their protective activity in a Winn assay. Figure 2 illustrates the results obtained after i.v. injection of 1 mg BCG-SpC; either the whole population, or the T-enriched (nylon-wool non-adherent) or T-depleted (anti $\theta$ treated) population.

In these particular experiments, the inhibition of the protective effect by previous injections of BCG-SpC was moderate. However, when BCG-SpC were injected after purification we observed an amplified inhibition by the T-enriched population and a loss of inhibition of protection by the T-depleted population (Figure 2).

In order to determine the phenotype of the T cells responsible for this suppression, T-enriched 1 mg BCG-SpC were treated with anti Lyt 1.2 or anti Lyt 2.2 antibodies +C. The transfer of the whole population as well as the T-enriched BCG-SpC actively suppressed the immunization of normal hosts (Figure 3). This suppression was found to be mediated by the Lyt 1 subpopulation of T cells since anti Lyt 1 treatment abrogated this suppressive effect, whereas anti Lyt 2 treatment had no effect (Figure 3).

*Effect of* in vivo *IL-2 administration on the suppressive activity induced by BCG pretreatment*

It has previously been assumed that a deficiency in the activity of helper factors might be a major cause of the reduced *in vitro* cytotoxicity developed by lymphocytes of BCG-treated mice (Kendall & Sabbadini, 1981). We attempted to test such a hypothesis as an explanation of the inhibitory effects on the antitumoral immune response observed after BCG treatment. In order to

**Table V** Effect of addition, in the Winn test, of spleen cells from normal or BCG-treated mice on the protection transferred by immune T lymphocytes

| | *No. of mice with tumour/total no. of mice*[a] *Spleen cells*[b] *from mice treated with the following doses of BCG* | |
|---|---|---|
| *Normal spleen cells*[b] | 0.05 *mg* | *1 mg* |
| 6/18 | 3/8 | 14/18[c] |

[a]Recorded on Day 30.
[b]$10^7$ spleen cells from normal or BCG-treated mice were added to $4 \times 10^6$ immune T cells and $10^4$ tumour cells (E:T=400:1).
[c]Significantly different ($P<0.02$) from control group receiving no BCG treatment.

**Figure 2** Evidence of suppressor cells induced by 1 mg BCG. B6 syngeneic mice received i.v. 2 h before AB2 immunization NSpC (■), BCG SpC either the whole population (▲) or T cell-depleted (△) or T cell-enriched (○) BCG SpC populations. Eight days later $5 \times 10^6$ immune T spleen cells derived from mice of the different groups were mixed with $10^4$ B6-1 tumour cells and injected s.c. to syngeneic B6 mice. Tumour growth of the mixtures was measured and compared to the growth of $10^4$ B6-1 tumour cells alone (●---●).

overcome this deficiency, we repeatedly administered *in vivo* exogenous IL-2, after BCG treatment. BCG, IL-2 and AB2 hybrid cells were given according to the protocol described in Materials and Methods. Protection against tumour growth obtained after immunization with AB2 hybrid cells was nearly abolished by pretreatment with 1 mg BCG and also, surprisingly after IL-2 repeated administration (Figure 4). The combination of BCG and IL-2 treatments decreased the inhibitory effect of BCG treatment.

### Discussion

The question addressed was whether BCG interferes with a specific *in vivo* antitumoral immune reaction and whether this interaction depends upon the dose of BCG and the state of immunization. The expression of active immunity conferred by an AB2 hybrid tumour cell inoculation was eliminated by a previous BCG injection, even at low doses. The mechanism responsible for tumour rejection in this model might be either nonspecific (NK, macrophages) or specific (immune T cells). The method of transferred immunity (Winn assay) circumvented the problem of nonspecific resistance which exists in actively immunized recipients and permitted the study of the effects of BCG on specific immunity mediated by T cells.

We have previously shown (Poupon *et al.*, 1979, 1981) that a specific response could be developed against tumour cells dissociated from MCA fibrosarcoma in B6 mice. Two types of immunizing protocols were used: the first consisted of repeatedly challenging B6 syngeneic mice with live tumour cells, followed by surgical excision of the tumour (Poupon *et al.*, 1979). The other consisted of a single injection of tumour cells hybridized with an allogeneic cell (Payelle *et al.*, 1981). These two different types of immunization resulted in an identical immune reaction characterized by a sharp specificity against the tumour cells and the transference of this immune protection only by T cells. Both types of immunization have been used in this study, depending on the question addressed.

Interactions between BCG treatment and the anti MC B6-1 antitumoral reaction were studied at different phases of the development of the immune state. The pretreatment of normal mice by low doses of BCG did not modify the level of the protective effect induced by injection of hybrid tumour cells. In some experiments, such BCG treatment even tended to result in a better response to the tumour antigen. A high dose of BCG (1 mg), however, dramatically inhibited the emergence of an immune response. Such a negative effect has previously been shown by many authors (Davies & Sabbadini, 1977; Florentin *et al.*, 1976; Turcotte *et al.*, 1978; Geffard & Orbach-Arbouys, 1976; Watson & Collins, 1980; Klimpel, 1979), and our results confirm this finding.

A further important question concerned the interference of BCG treatment with an established antitumoral immune state. We demonstrated that BCG effects were complex. At low doses (0.01 mg), BCG had a potentiating effect on the immune response. In contrast, a high dose of BCG (1 mg) completely abolished the pre-existing immunity. This was shown both by the loss of the protective effect of T lymphocytes originating from previously immunized mice, and by the death of the immunized mice following rapid tumour growth; a phenomenon which is not normally observed at this stage of immunization. When the small dose was repeated, the benefit was lost. This might be linked

**Figure 3** Characterization of suppressor cells induced by 1 mg BCG. B6 syngeneic mice received 2 h before AB2 immunization N SpC (■), BCG SpC either the whole population (▲), or T cell-enriched (○) or T cell-enriched anti Lyt 1-treated (□) or T cell-enriched anti Lyt 2-treated (△) BCG SpC populations. Eight days later $5 \times 10^6$ immune T spleen cells derived from mice of the different groups were mixed with $10^4$ B6-1 tumour cells and injected s.c. to syngeneic B6 mice. Tumour growth of the mixtures was measured and compared to the growth of $10^4$ B6-1 tumour cells alone (●---●).

to the progressive development of BCG colonies in the spleen of the treated mice.

In our experiment, two administrations of low doses of BCG at a 22-day interval followed by tumour challenge 10 days later, resulted in an elevated number of bacilli that was more rapidly obtained in the case of a high dose of BCG. Therefore, it is not surprising that similar inhibitory effects were observed in both situations if these effects are linked to the development of BCG colonies. Several possibilities to explain inhibitory effects have to be considered: (i) production of BCG-induced-blocking factors which enhance tumour growth. These factors have been demonstrated in different tumour systems both in mice (Hawrylko, 1977) and rats (Bansal *et al.*, 1973). (ii) The induction of suppressor cells which are active at the effector phase of the specific antitumoral protection. Our results suggested the presence, in the spleen of mice treated with high dose BCG, of suppressor cells active on the effector phase of the specific antitumoural response. We demonstrated more directly the presence of suppressor cells which develop after BCG treatment by a direct effect on immune activity through mixing BCG suppressor cells with immune lymphocytes. These experiments clearly showed the inhibitory effect of BCG-SpC on the protective effect of transferred immune T cells. A second phase of these studies demonstrated that suppression also acts at the induction stage of immunization. We showed a suppression of the generation of immune T cells mediated by nylon-non adherent cells derived from 1 mg BCG treated mice. This suppression was eliminated by the treatment of BCG-SpC with anti Thy 1.2 +C before the transfer. Treatment with anti Lyt 1.2 + C

**Figure 4** Effect of IL-2 supplementation on the suppressive activity of BCG pretreatment. Growth of tumours in mice receiving tumour cells alone (●), or mixed with immune T cells (■), or with T cells derived from BCG (1 mg) treated immune mice (▲), or with T cells derived from IL-2 supplemented immune mice (□) or with T cells derived from BCG (1 mg) pretreated IL-2 supplemented immune mice (△).

eliminated the suppressor activity of the BCG-T cell population whereas treatment with anti Lyt 2.2 + C did not affect the suppression. Therefore, the suppression induced by high dose BCG is mediated by Lyt $1^+2^-$ T cells. It was recently demonstrated that antigen nonspecific suppression of cytotoxic T lymphocyte responses to alloantigen is also mediated by a T cell of Ly $1^+2^-$ phenotype (Hathcock & Hodes, 1983). The presence of suppressor macrophages was shown to be operative, using mainly *in vitro* systems (Shrier *et al.*, 1980; Klimpel *et al.*, 1979; Wadee *et al.*, 1980). Suppressor T cells have been implicated in the inhibition of DTH reactions (Nakamura *et al.*, 1982; Kato & Yamamoto, 1982; Watson & Collins, 1980), lymphoproliferative responses (Florentin *et al.*, 1976) and GVH reactivity (Geffard & Orbach-Arbouys, 1976).

Several mechanisms of T cell mediated suppression have bee postulated by the different workers: (i) an increased absorption of helper factors by the suppressor cells (Klimpel *et al.*, 1979); (ii) an alteration of the kinetics of the interaction between the cytotoxic cells and their targets during the T cell mediated cytotoxicity (Davies & Sabbadini, 1978); (iii) an inhibition of the capacity of Lyt $1^+$ T cells to produce helper factors (Klimpel *et al.*, 1979; Kendall & Sabbadini, 1981), since *in vitro* addition of exogenous IL-2 completely restored the response. Kendall &

Sabbadini (1981) suggested two hypotheses for the reduced capacity of Lyt $1^+$ T cells to produce IL-2; the direct action of BCG on these Lyt $1^+$ cells, or the mediation of BCG-induced suppressor cells.

Combined BCG and IL-2 treatment was followed by delayed tumour growth as compared to BCG given alone. But, since IL-2 given repeatedly to immunized mice abrogated the protection, the interaction between the effects of IL-2 and BCG appears very complex and interpretation of these results is difficult. Our results clearly demonstrate that T suppressor cells of the Ly $1^+2^-$ phenotype are responsible, at least partially, for the reduced response induced by high dose BCG. Other mechanisms, beside the intervention of a suppressive activity, have been demonstrated to be implicated in the reduced response of BCG-treated animals: a reduced frequency of CTL precursors (Kendall & Sabbadini, 1981), an acceleration of the destruction of antigen (Perkins & Makinodan, 1965), a blockade of the RES (Sabet *et al.*, 1969).

This work confirms the complex effects of BCG, which may be beneficial or harmful to the immunological control of tumour growth, depending upon its mode of application, and especially on the dosage used. We demonstrated that BCG induces T suppressor cells, active on the afferent and on the efferent arm of the anti-tumour immune response. BCG also stimulates macrophages with antitumour activity. These contradictory activities are important to consider when using BCG in cancer therapy.

This work was supported by grant from INSERM (CRL 79.5.516.2). We are indebted to Dr Chaouat for the gift of IL-2. We thank V. Chanoina and I. Vergnon for their excellent technical assistance.

### References

BALDWIN, R., HOPPER, D.G. & PIMM, M.W. (1976). Bacillus Calmette-Guérin contact immunotherapy of local and metastatic deposits of rat tumors. *Ann. N.Y. Acad. Sci.*, **277**, 124.

BANSAL, S.C. & SJOGREN, H.O. (1973). Regression of polyoma tumor metastasis by combined unblocking and BCG treatment. Correlation with induced alterations in tumor immunity status. *Int. J. Cancer*, **12**, 179.

DAVIES, M. & SABBADINI, E. (1977). Effect of different doses of BCG on an allogeneic cell-mediated anti-tumour response in mice. *J. Natl Cancer Inst.*, **59**, 263.

DAVIES, M. & SABBADINI, E. (1978). Inhibition of cell-mediated cytotoxicity by plastic-adherent cells in the spleens of mice treated with BCG and reacting to an allogeneic tumor. *Fed. Proc.*, **37**, 1353.

FLORENTIN, I., HUCHET, R., BRULEY-ROSSET, M., HALLE-PANNENKO, O. & MATHE, G. (1976). Studies on the mechanisms of action of BCG. *Cancer Immunol. Immunother.*, **1**, 31.

GEFFARD, J.S. & ORBACH-ARBOUYS, S. (1976). Enhancement of T suppressor activity in mice by high doses of BCG. *Cancer Immunol. Immunother.*, **1**, 41.

HATHCOCK, S.K. & HODES, R.J. (1983). Regulatory mechanisms in cell-mediated immune responses. V-Distinct Lyt subsets mediate antigen-specific and antigen nonspecific suppression. *Transplantation*, **36**, 298.

HAWRYLKO, E. (1977). Serum-mediated escape from BCG-potentiated antitumor immunity *in vivo*. *J. Natl Cancer Inst.*, **59**, 367.

JULIUS, M.H., SIMPSON, E. & HERZENBERG, L.A. (1973). A rapid method for the isolation of functional thymus-derived murine lymphocytes. *Eur. J. Immunol.*, **3**, 645.

KATO, K. & YAMAMOTO, K. (1982). Suppression of BCG cell wall-induced delayed-type hypersensitivity by BCG pretreatment. II – Induction of suppressor T cells by heat-killed BCG injection. *Immunology*, **45**, 655.

KENDALL, L. & SABBADINI, E. (1981). Effect of bacillus Calmette-Guérin on the *in vitro* generation of cytotoxic T lymphocytes. I – Effect of BCG on the frequency of cytotoxic T lymphocyte precursors and on the production of helper factors. *J. Immunol.*, **127**, 234.

KITAMURA, Y., NOMOTO, K., TORISU, M. & TAKEYA, K. (1976). Effects of BCG (Bacillus Calmette-Guérin) vaccines on immune responses in mice. I – Possible effect of BCG on helper T cells. *Japan J. Microbiol.*, **20**, 303.

KLIMPEL, G.R. & HENNEY, C.S. (1978). BCG-induced suppressor cells. I – Demonstration of a macrophage-like suppressor cell that inhibits cytotoxic T cell generation *in vitro*. *J. Immunol.*, **120**, 563.

KLIMPEL, G.R. (1979). Soluble factors from BCG-induced suppressor cells inhibit *in vitro* PFC responses but not cytotoxic responses. *Cell. Immunol.*, **47**, 218.

KLIMPEL, G.R., ODAKA, M. & HENNEY, C.S. (1979). Inhibition of *in vitro* cytotoxic responses by BCG-induced macrophage-like suppressor cells. II – Suppression occurs at the level of a "helper" T cell. *J. Immunol.*, **123**, 350.

NAKAMURA, R.M., TANAKA, H. & TOKUNAGA, T. (1982). Induction of suppressor T cells in delayed-type hypersensitivity to mycobacterium bovis BCG low responder mice. *Infect. Immun.*, **28**, 331.

OLD, L.J., CLARKE, D.A. & BENACERRAF, B. (1959). Effect of Bacillus Calmette-Guérin infection on transplanted tumors in the mouse. *Nature*, **184**, 291.

PAYELLE, B., POUPON, M.F. & LESPINATS, G. (1981). Adoptive transfer of immunity induced by semi-allogeneic hybrid cells, against a murine fibrosarcoma. *Int. J. Cancer*, **27**. 783.

PERKINS, L.H. & MAKINODAN, T. (1965). The suppressive role of mouse peritoneal phagocytes in agglutinin response. *J. Immunol.*, **94**, 765.

POUPON, M.F., LESPINATS, G., KOLB, J.P. & PAYELLE, B. (1979). Immunity to 3-methylcholanthrene-induced fibrosarcoma in the C57BL/6 mouse: *in vivo* analysis by adoptive tumor neutralization test. *J. Natl Cancer Inst.*, **62,** 989.

POUPON, M.F., PAYELLE, B. & LESPINATS, G. (1981). Adoption of tumor immunity: role of the major histocompatibility complex. *J. Immunol.*, **126,** 2342.

SABET, T., NEWLIN, C. & FREIDMAN, H. (1969). Effects of RES blockade on antibody formation. I – Suppressed cellular and humoral haemolysis responses in mice injected with carbon particles. *Immunology,* **16,** 433.

SCHRIER, D.J., ALLEN, E.M. & MOORE, V.L. (1980). BCG-induced macrophage suppression in mice: suppression of specific and nonspecific antibody-mediated and cellular immunologic responses. *Cell. Immunol.*, **56,** 347.

TURCOTTE, R., LAFLEUR, L. & LABRECHE, M. (1978). Opposite effects of BCG on spleen and lymph node cells: lymphocyte proliferation and immunoglobulin synthesis. *Infect. Immunol.*, **21,** 696.

WADEE, A.A., SHER, R. & RABSON, A.R. (1980). Production of a suppressor factor by human adherent cells treated with mycobacteria. *J. Immunol.*, **125,** 1380.

WATSON, S.R. & COLLINS, F.M. (1980). Development of suppressor T cells in mice heavily infected with mycobacteria. *Immunology,* **39,** 367.

WINN, H.T. (1961). Immune mechanisms in homo transplantation. II – Quantitative assay of immunologic activity of lymphoid cells stimulated by tumor homografts. *J. Immunol.*, **86,** 228.

ZBAR, B., BERNSTEIN, I., TANAKA, T. & RAPP, H. (1970). Tumor immunity produced by the intradermal inoculation of living Mycobacterium bovis (strain BCG). *Science,* **170,** 1217.


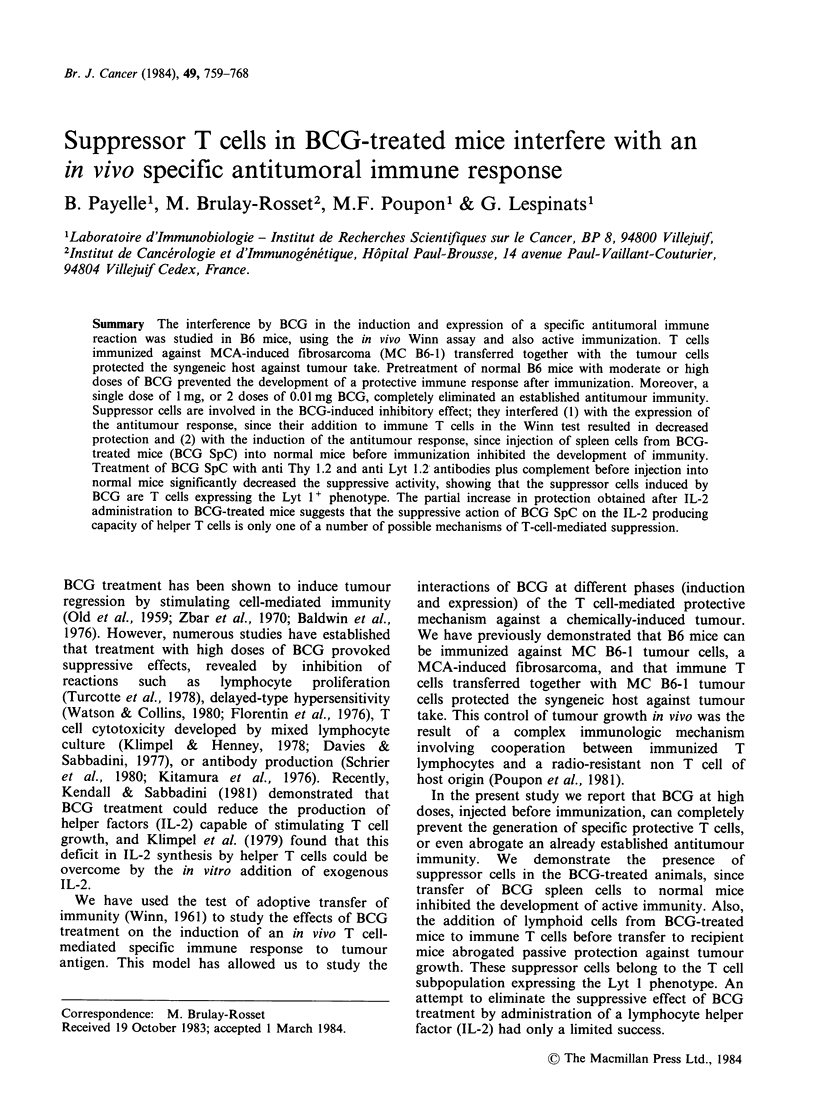

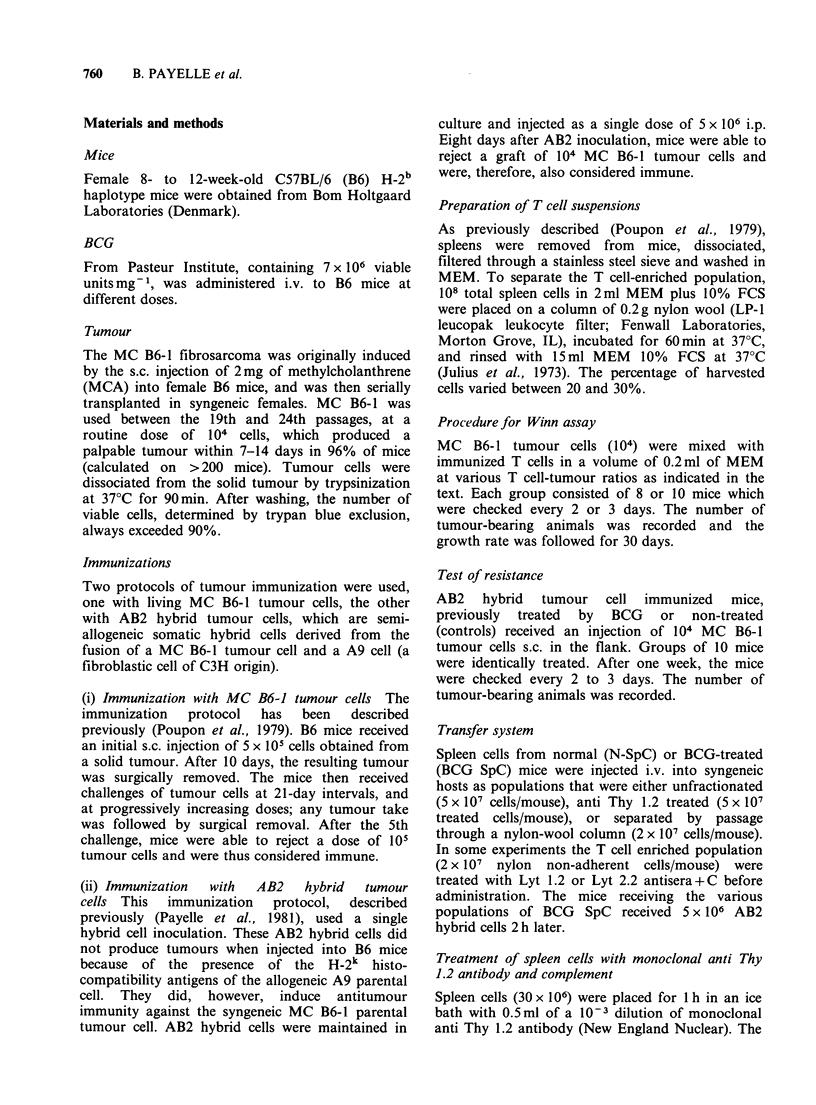

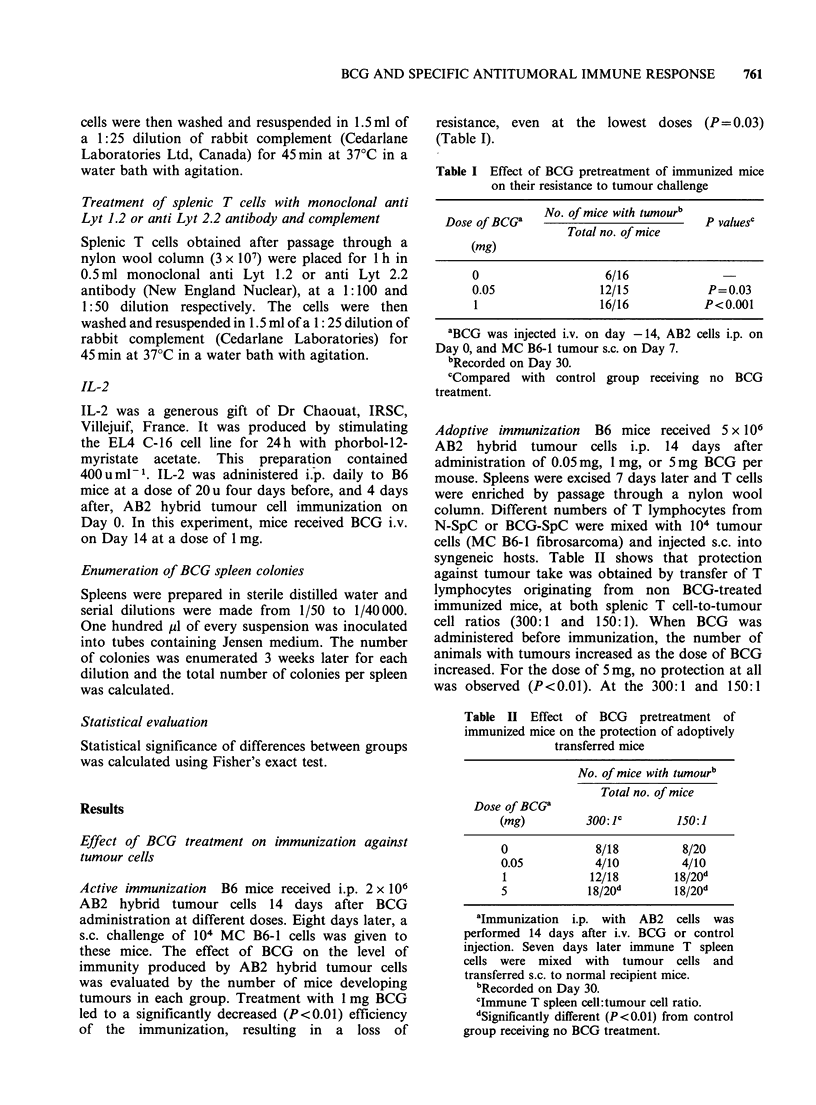

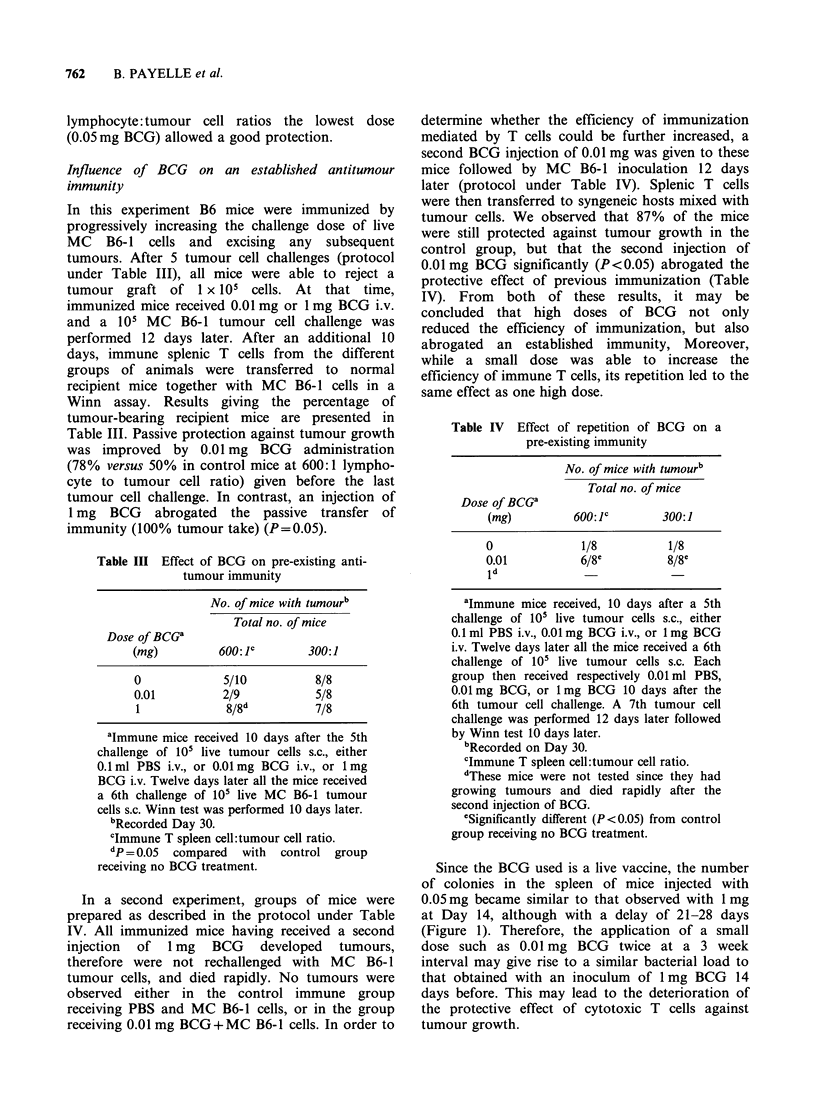

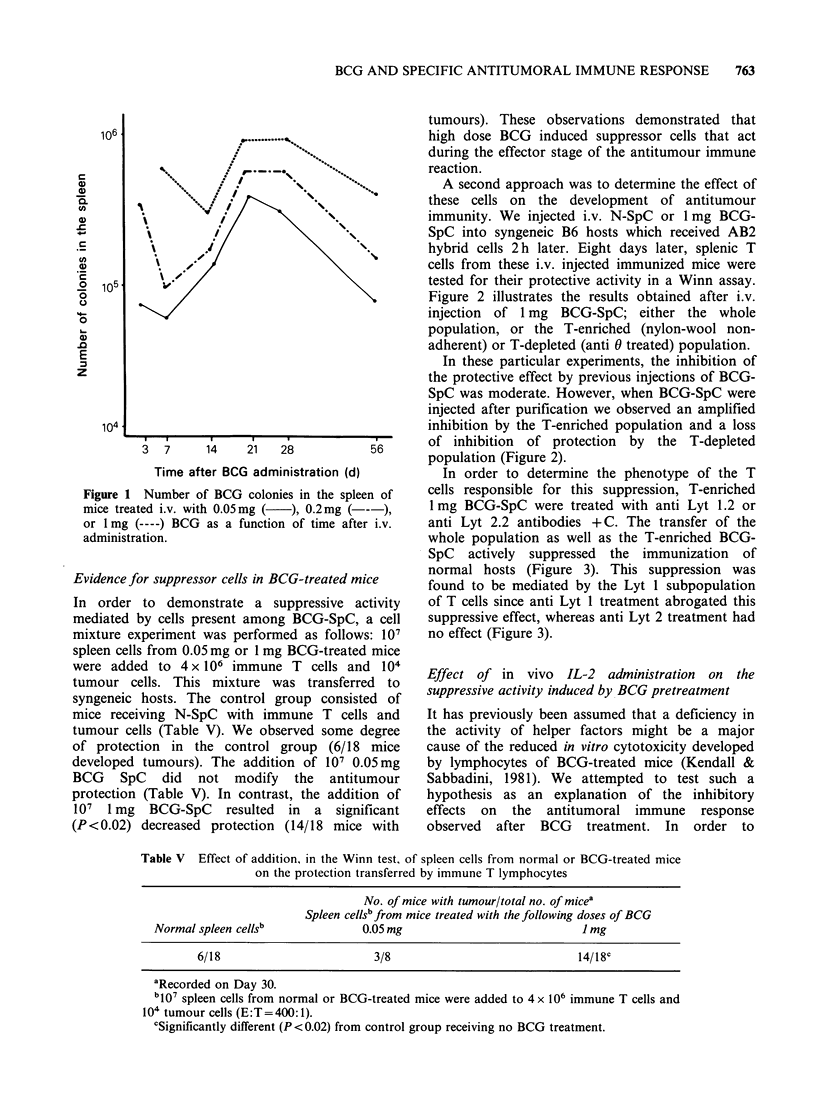

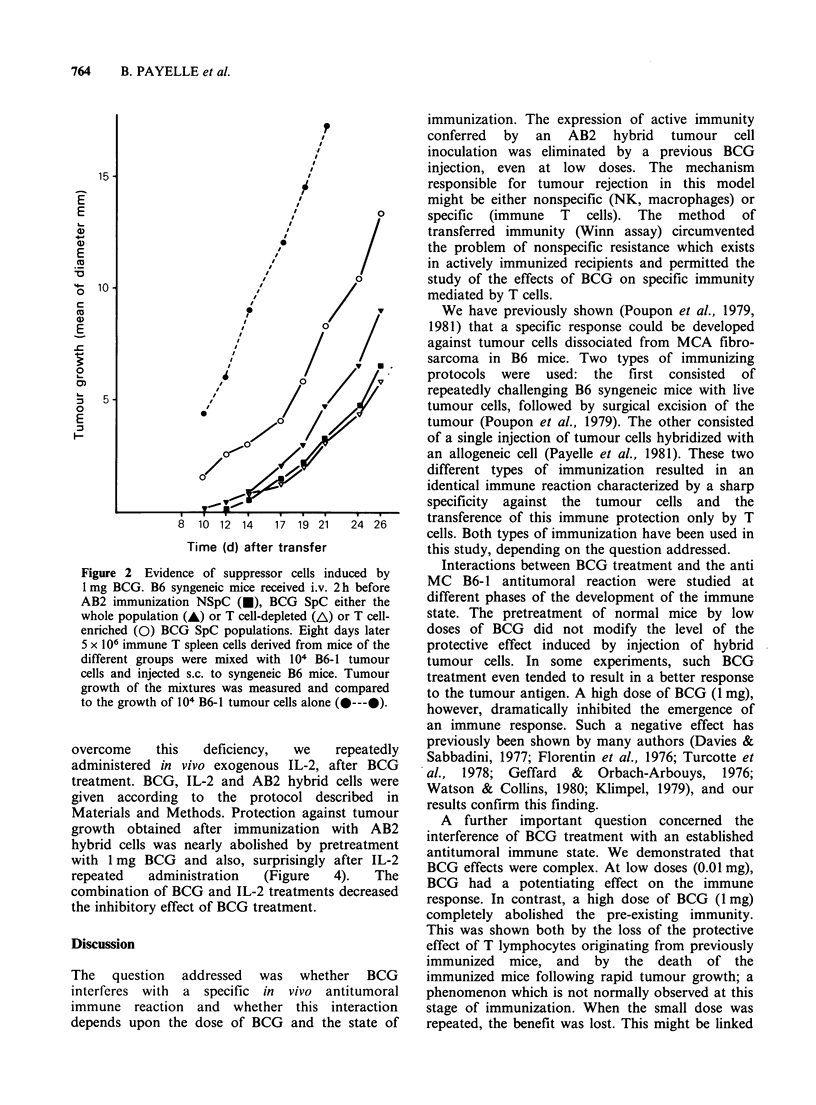

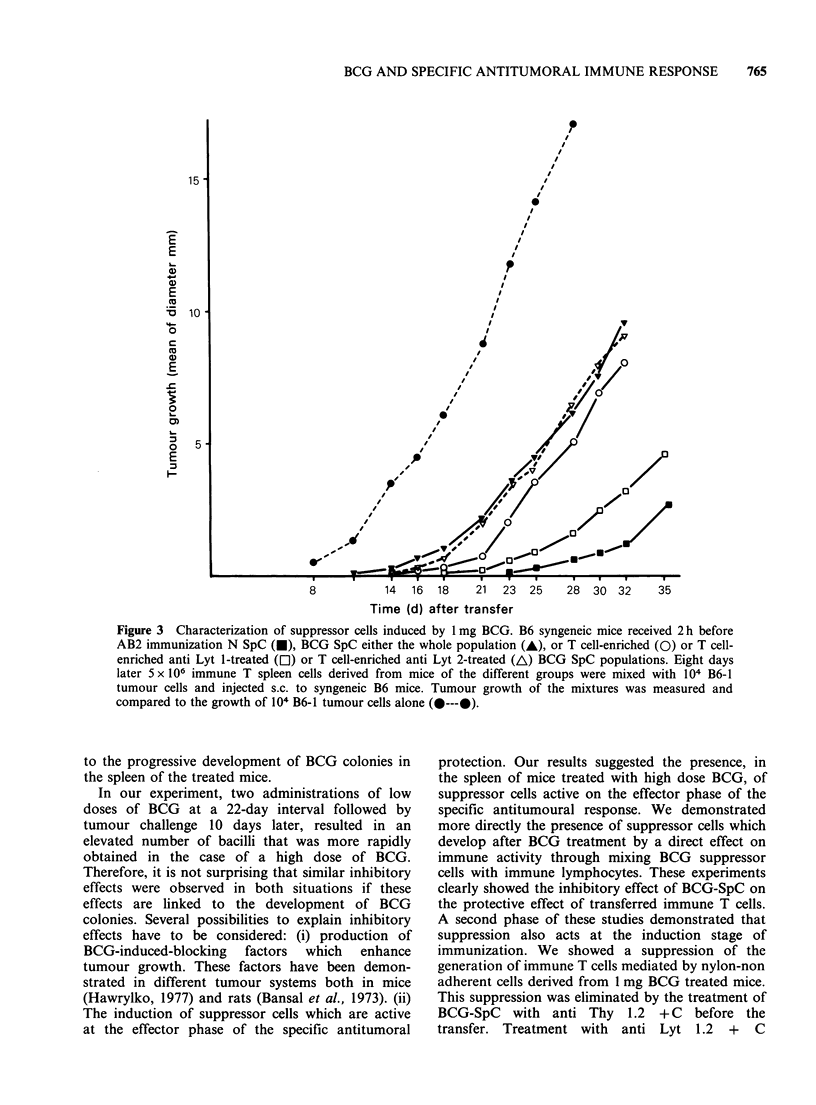

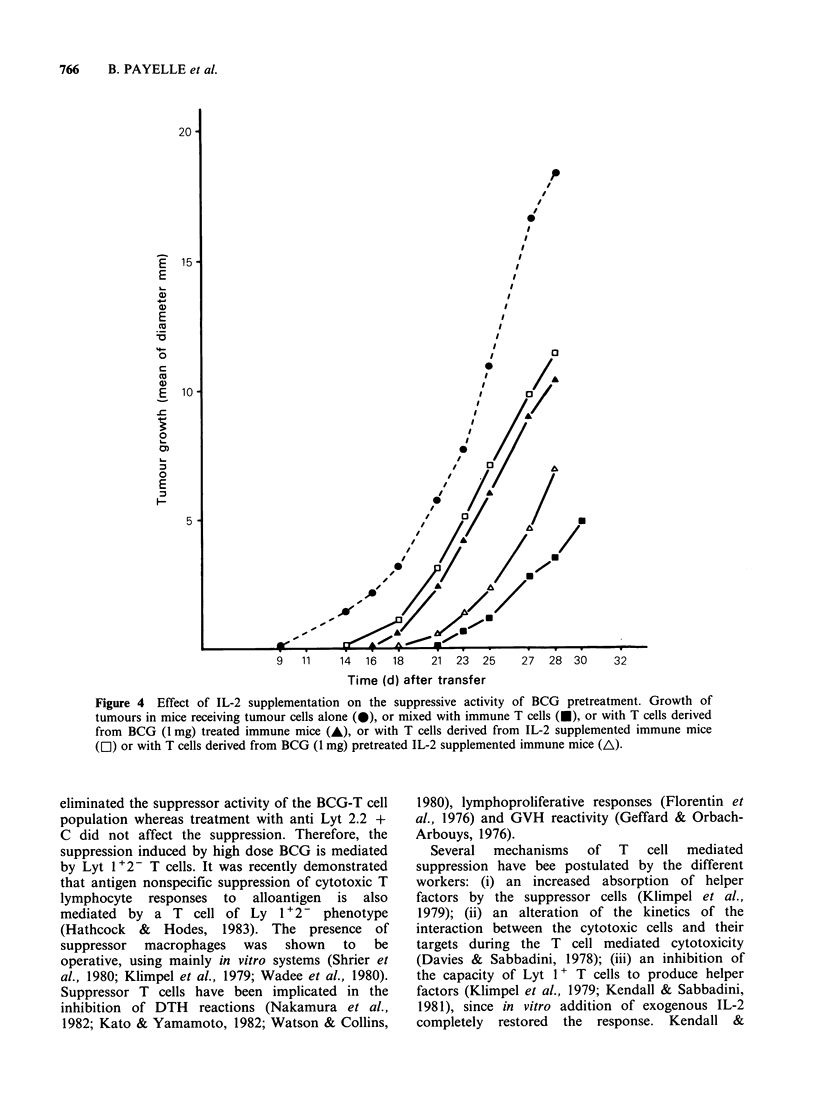

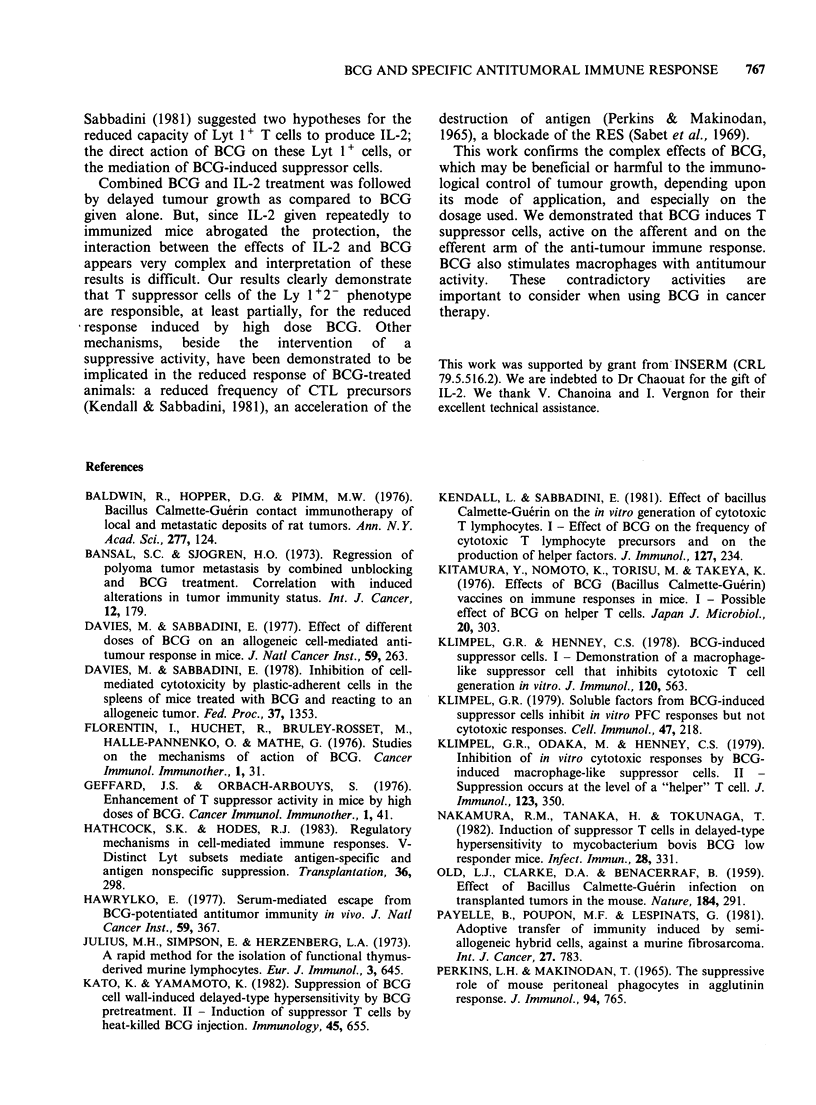

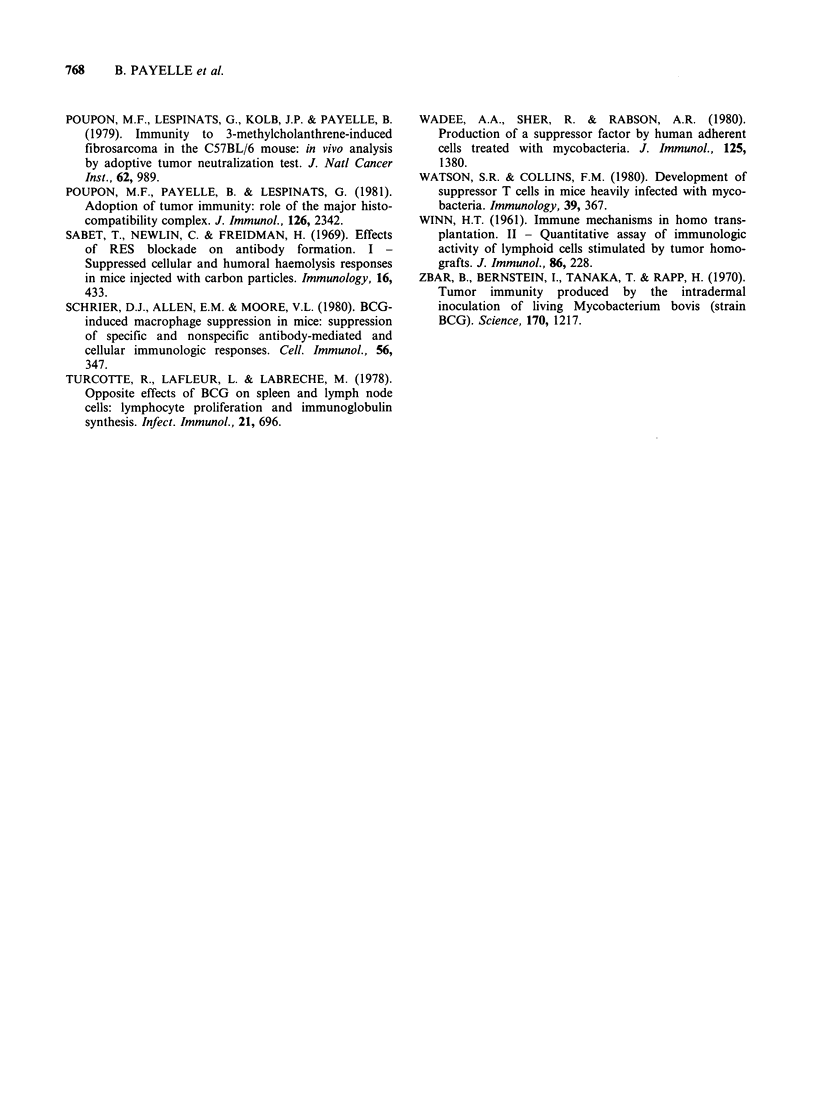


## References

[OCR_01009] Baldwin R. W., Hopper D. G., Pimm M. V. (1976). Bacillus Calmette-Guérin contact immunotherapy of local and metastatic deposits of rat tumors.. Ann N Y Acad Sci.

[OCR_01015] Bansal S. C., Sjögren H. O. (1973). Regression of polyoma tumor metastasis by combined unblocking and BCG treatment--correlation with induced alterations in tumor immunity status.. Int J Cancer.

[OCR_01022] Davies M., Sabbadini E. (1977). Effect of different doses of BCG on an allogeneic cell-mediated antitumor response in mice.. J Natl Cancer Inst.

[OCR_01044] Hathcock K. S., Hodes R. J. (1983). Regulatory mechanisms in cell-mediated immune response. V. Distinct Lyt subsets mediate antigen-specific and antigen-nonspecific suppression.. Transplantation.

[OCR_01056] Julius M. H., Simpson E., Herzenberg L. A. (1973). A rapid method for the isolation of functional thymus-derived murine lymphocytes.. Eur J Immunol.

[OCR_01061] Kato K., Yamamoto K. (1982). Suppression of BCG cell wall-induced delayed-type hypersensitivity by BCG pre-treatment. II. Induction of suppressor T cells by heat-killed BCG injection.. Immunology.

[OCR_01067] Kendall L., Sabbadini E. (1981). Effect of Bacillus Calmette-Guérin on the in vitro generation of cytotoxic T lymphocytes. I. Effect of BCG on the frequency of cytotoxic T lymphocyte precursors and on the production of helper factors.. J Immunol.

[OCR_01074] Kitamura Y., Nomoto K., Torisu M., Takeya K. (1976). Effects of BCG (Bacillus Calmette-Guérin) vaccines on immune responses in mice. I. Possible effect of BCG on helper T cells.. Jpn J Microbiol.

[OCR_01081] Klimpel G. R., Henney C. S. (1978). BCG-induced suppressor cells. I. Demonstration of a macrophage-like suppressor cell that inhibits cytotoxic T cell generation in vitro.. J Immunol.

[OCR_01092] Klimpel G. R., Okada M., Henney C. S. (1979). Inhibition of in vitro cytotoxic responses by BCG-induced macrophage-like suppressor cells. II. Suppression occurs at the level of a "helper" T cell.. J Immunol.

[OCR_01087] Klimpel G. R. (1979). Soluble factors from BCG-induced suppressor cells inhibit in vitro PFC responses but not cytotoxic responses.. Cell Immunol.

[OCR_01099] Nakamura R. M., Tokunaga T. (1980). Induction of suppressor T cells in delayed-type hypersensitivity to Mycobacterium bovis BCG in low-responder mice.. Infect Immun.

[OCR_01105] OLD L. J., CLARKE D. A., BENACERRAF B. (1959). Effect of Bacillus Calmette-Guerin infection on transplanted tumours in the mouse.. Nature.

[OCR_01116] PERKINS E. H., MAKINODAN T. (1965). THE SUPPRESSIVE ROLE OF MOUSE PERITONEAL PHAGOCYTES IN AGGLUTININ RESPONSE.. J Immunol.

[OCR_01110] Payelle B., Poupon M. F., Lespinats G. (1981). Adoptive transfer of immunity induced by semi-allogeneic hybrid cells, against a murine fibrosarcoma.. Int J Cancer.

[OCR_01123] Poupon M. F., Lespinats G., Kolb J. P., Payelle B. (1979). Immunity to a 3-methylcholanthrene-induced fibrosarcoma in the C57BL/6 mouse: in vivo analysis by the adoptive tumor neutralization test.. J Natl Cancer Inst.

[OCR_01130] Poupon M. F., Payelle B., Lespinats G. (1981). Adoption of tumor immunity: role of the major histocompatibility complex.. J Immunol.

[OCR_01135] Sabet T., Newlin C., Friedman H. (1969). Effects of RES "blockade" on antibody-formation. I. Suppressed cellular and humoral haemolysin responses in mice injected with carbon particles.. Immunology.

[OCR_01142] Schrier D. J., Allen E. M., Moore V. L. (1980). BCG-induced macrophage suppression in mice: suppression of specific and nonspecific antibody-medicated and cellular immunologic responses.. Cell Immunol.

[OCR_01149] Turcotte R., Lafleur L., Labrèche M. (1978). Opposite effects of BCG on spleen and lymph node cells: lymphocyte proliferation and immunoglobulin synthesis.. Infect Immun.

[OCR_01166] WINN H. J. (1961). Immune mechanisms in homotransplantation. II. Quantitative assay of the immunologic activity of lymphoid cells stimulated by tumor homografts.. J Immunol.

[OCR_01155] Wadee A. A., Sher R., Rabson A. R. (1980). Production of a suppressor factor by human adherent cells treated with mycobacteria.. J Immunol.

[OCR_01161] Watson S. R., Collins F. M. (1980). Development of suppressor T cells in mice heavily infected with mycobacteria.. Immunology.

[OCR_01172] Zbar B., Bernstein I., Tanaka T., Rapp H. J. (1970). Tumor immunity produced by the intradermal inoculation of living tumor cells and living Mycobacterium bovis (strain BCG).. Science.

